# Effects of intensive care unit ambient sounds on healthcare professionals: results of an online survey and noise exposure in an experimental setting

**DOI:** 10.1186/s40635-020-00321-3

**Published:** 2020-07-23

**Authors:** Nadine Schmidt, Stephan M. Gerber, Björn Zante, Tom Gawliczek, Alvin Chesham, Klemens Gutbrod, René M. Müri, Tobias Nef, Joerg C. Schefold, Marie-Madlen Jeitziner

**Affiliations:** 1 Gerontechnology & Rehabilitation Group, University of Bern, Murtenstrasse 50, CH-3008 Bern, Switzerland; 2 Department of Intensive Care Medicine, University Hospital Bern (Inselspital), University of Bern, CH-3010 Bern, Switzerland; 3 Hearing Research Laboratory, University of Bern, Murtenstrasse 50, CH-3008 Bern, Switzerland; 4 Department of Neurology, University Neurorehabilitation, University Hospital Bern (Inselspital), University of Bern, Freiburgstrasse, CH-3010 Bern, Switzerland; 5 ARTORG Center for Biomedical Engineering Research, University of Bern, Murtenstrasse 50, CH-3008 Bern, Switzerland

**Keywords:** Intensive care unit, Noise, Healthcare professionals, Working memory, Annoyance, Performance

## Abstract

**Background:**

Noise levels on intensive care units (ICUs) are typically elevated. While many studies reported negative effects of ICU ambient sounds on patients, only few investigated noise as a factor to influence well-being or performance in healthcare professionals.

**Methods:**

An online survey in the German-speaking part of Switzerland was conducted to assess how ICU soundscapes are subjectively perceived by healthcare professionals. The questionnaire was answered by 348 participants. Additionally, effects of noise on working memory performance were evaluated in an experimental noise exposure setting. Twenty-six healthcare professionals and 27 healthy controls performed a 2-back object-location task while being exposed to either ICU or pink noise.

**Results:**

Survey results demonstrate that a majority of participants was aware of heightened noise levels. Participants reported that mostly well-being, performance, and attention could be reduced, along with subjective annoyance and fatigue by ICU ambient sounds. Although no significant effects of noise exposure on working memory performance was observed, self-assessments revealed significantly higher stress levels, increased annoyance and distraction ratings as well as decreased confidence in performance after ICU-noise exposure.

**Conclusion:**

Subjective assessments indicate that heightened noise levels on ICUs induce annoyance, with heightened stress levels, impaired well-being, and reduced performance being potential consequences. Empirical evidence with objective and physiological measures is warranted.

## Background

Noise is known to exert negative effects on human beings [1, 2]. Also in the hospital context, studies showed that sleep quality of patients might be impaired due to ambient sounds [3]. Therefore, the World Health Organization (WHO) recommended 20 years ago that sound pressure levels in intensive care units (ICUs) should not exceed 35 dBA [1]. Since then, several studies demonstrated that this guideline can hardly be met [4–8]. Exceeding noise levels do not only seem to affect patients but also healthcare professionals: Studies showed that noise on ICUs contributes to annoyance, irritation, fatigue, stress, and occupational burnout symptoms of healthcare professionals (HCPs) [9–13]. Moreover, in one study noise was named to be a considerable performance obstacle [14]. Nonetheless, studies about effects of ICU noise on cognition and working performance are sparse. More studies addressed cognition and working performance in operating rooms (ORs): In a survey, a majority of participating surgical healthcare professionals reported that communication with colleagues and concentration is impaired by OR ambient sounds with errors being more likely [15]. In a study of Murthy et al. mean short-term and working memory performance of 20 anesthesia residents was significantly lower when exposed to OR noise compared to when exposed to no ambient sounds [16]. Also, outside the hospital context, ambient noise was found to have detrimental effects for individuals [17, 18]. These effects seem to be dependable on specific acoustical characteristics (e.g., loudness or frequency) and non-acoustical characteristics of the sound (e.g., content or meaning) [19, 20].

No current data are available on healthcare professionals’ awareness and perception of ICU ambient sounds. Studies investigating possible effects of ICU noise in an experimental setting are wanted. The aim of this study was to assess possible well-being and health-related factors that might be affected by ICU noise. To this end, an online survey as well as an experiment was conducted.

## Methods

The aim of the online survey was to assess whether healthcare professionals in German-speaking Switzerland are aware of increased sound levels on ICUs and whether they perceive noise as a strain-inducing factor. Further, different noise sources in ICUs (such as different alarms and equipment sounds) were examined according to perceived irritation and sound levels.

The aim of the noise exposure experiment was to evaluate potential effects of ICU-noise exposure on cognitive performance and on different self-rated variables including stress, distraction, performance, or annoyance.

### Design and materials

For the online survey, a 17-item questionnaire was used based on a previously developed questionnaire by Ryherd, Waye, and Ljungkvist [13]: The items were translated to German and formulated as questions. Further, some of the items were left out, others were added (see Fig. 2). Questions were answered on a four-point Likert scale (1 = “no”; 2 = “rather no”; 3 = “rather yes”, 4 = “yes”). Additionally, 20 ICU-noise sources were listed (see Table 1 for noise sources). Each source was rated according to its perceived irritation on a 4-point scale (from 1 = “not at all irritating” to 4 = “very irritating”). An estimation of dBA level was requested with provision of five reference values (“0 dBA: hearing threshold; 25 dBA: breathing sound; 50 dBA: bird twittering; 75 dBA: car; 100 dBA: circular saw”).

**Table 1:** Irritation ratings and sound level estimations of 20 noise sources per category

	Irritation rating			dBA estimation			Sound level (in dBA)
Noise source	Mean ± SD	Rank		Mean ± SD	Rank		
Telephone	3.26 ± 0.81	1		63.4 ± 13.7	3		70-80 [28]
Surveillance monitor (alarms)	3.21 ± 0.72	2		67.0 ± 13.1	2		44-78 [29]
Conversation of colleagues	3.10 ± 0.82	3		63.3 ± 14.6	4		59-90 [29]
Open packages	3.08 ± 1.01	4		68.4 ± 19.2	1		86 [29]
Pager	2.92 ± 0.98	5		58.9 ± 18.7	9		84 [28]
Dialysis machine	2.75 ± 0.89	6		60.3 ± 18.8	7		55 [a]
Mechanical ventilators	2.74 ± 0.83	7		60.7 ± 15.8	6		49-77 [7]
Squeaking shoes	2.68 ± 0.97	8		52.4 ± 18.8	17		
Medical visit	2.58 ± 0.92	9		59.8 ± 15.4	8		59-90 [29]
Syringe pump	2.57 ± 0.85	10		55.1 ± 19.4	14		
Suction pump	2.47 ± 0.89	11		55.6 ± 16.5	11		70-82 [7] (open)
Transport monitor, ventilator	2.41 ± 0.82	12		60.7 ± 16.6	5		
Ringing/bell	2.39 ± 0.94	13		55.2 ± 18.2	12		40 [a]
ECMO machine	2.36 ± 1.02	14		56.4 ± 20.3	10		
Open/close drawers	2.30 ± 0.88	15		52.7 ± 19.1	16		85.7 [7]
Conversation of visitors	2.29 ± 0.76	16		53.8 ± 15.2	15		59-90 [29]
Brake on the bed	2.18 ± 0.98	17		55.1 ± 21.2	13		
Heated blanket	2.16 ± 0.92	18		44.0 ± 18.9	19		40 [a]
Compressed air	2.10 ± 0.93	19		51.7 ± 20.4	18		70-77 [7] (open)
Thoracic drainage	1.60 ± 0.69	20		36.2 ± 19.2	20		45 [a]

*dBA* A-weighted decibel scale, *ECMO* extracorporeal membrane oxygenation

Sources: [a] measurements by authors of this study [7]; Tsiou, Eftymiatos, Theodossopoulou, Notis, and Kiriakou, 1998 [28]; Sommargren, 1995 [29]; Pugh, 2007; blanks: no measures available

**Fig. 1:**
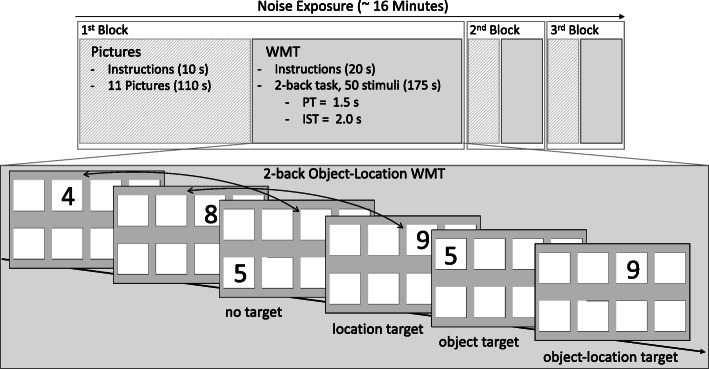
Procedure of the experimental noise exposure. One session consisted of three blocks with each block being divided into a pictures-viewing part and a working memory task. Participants had to press a yes button if either the current number and/or the box was the same as two steps before (targets). If not, a no button had to be pressed. WMT, working memory task; PT, presentation time; IST, interstimulus time

The experimental setting noise exposure was performed with the grouping variable (ICU-healthcare professional HCP/ controls) as between-subject variation and noise condition (ICU noise/pink noise) as within-subject variation. A record of a cardiopulmonary resuscitation (CPR) simulation in an ICU environment (55-85 dBA) was chosen as the ICU-noise condition, whereas pink noise served as baseline condition (35 dBA). Pink noise is a random sound containing all frequencies but with decreasing power over the frequency spectrum. Studies suggest that pink noise—in contrast to other ambient sounds—has no negative effect on cognitive performance [21, 22]. All participants attended in two sessions (range 12 to 72 days). Half of them were tested under ICU noise in the first, and pink noise in the second session whereas the other half was exposed to the ICU noise first. Stratified randomization based on the groups to assign the timepoint of condition was used. The procedure of the experimental noise exposure is explained in Fig. 1. One session was divided into three parts, each part consisting of a rest-phase where pictures—randomly chosen out of the OASIS [23]—were shown, and of a two-back object-location working memory task (WMT). In the WMT, different digits appeared consecutively on different locations of the screen and participants had to answer via a response box whether the current digit was a target (same object or same location as two steps before) or not. Overall, subjects were exposed for approximately 16 min to the noise.

Before and after noise exposure, questionnaires were assessed: Besides demographic information, German versions of the Weinstein Noise Sensitivity-Scale (WNS) [24, 25] and the Morningness-Eveningness-Questionnaires (D-MEQ) [26] were conducted in the beginning of the first session. Further, in both sessions, a sleep quality questionnaire (SF-A/R [27]), as well as questions on caffeine alcohol, and drug use were conducted. A visual scale was used to assess participants’ self-rated stress level and one for self-rated alertness. In both sessions, the task was followed by a questionnaire including perceived stress (0 “not at all stressful” to 100 “very stressful”), performance, annoyance, and distraction (1 “not at all” to 4 “strongly”) (see Table 3 for questions).

### Participants

The web link to the questionnaire was distributed via email to different ICUs from the German-speaking part of Switzerland. Participants were motivated to distribute the link among their colleagues. The questionnaire was accessible from 12 March to 19 June 2019.

Participants for the experimental study part were recruited at the University Hospital Bern and directly contacted by email. All participants had normal or, in one case, corrected-to-normal hearing with a hearing device.

### Statistical analysis

Relative proportions of the given answers of the first online survey part were evaluated. For the irritation ratings and estimations of dBA levels, descriptive statistics were used. To evaluate whether estimations of dBA were linked to irritation ratings, a linear regression model over all 20 noise sources was conducted. Other sources that were named in a free text box were gathered.

Independent-two-samples *t*-tests for difference in mean age between groups and conditions were conducted for the noise exposure experiment. Due to lack of normal distribution, strain and alertness ratings were analyzed with the non-parametric Wilcoxon rank-sum test for paired measurements to check for differences between conditions. Mixed analysis of variances (ANOVAs) with noise condition as a within and group membership as a between variable were computed to analyze differences in sleep quality. A two-way between ANOVA for Weinstein noise sensitivity scores was conducted to detect possible differences between HCPs and controls as well as between noise conditions. Further, the effect of age and of the dummy-coded grouping variable (controls = 0) on noise sensitivity was evaluated with a multiple linear regression model.

As outcome variables of the WMT, a performance and an accuracy measure were calculated. *Performance* was calculated as ratio of correct answers to all items and thus included no responses whereas *accuracy* refers to the specificity of a given answer by calculating the ratio of correct answers to all given answers, with no responses being ignored.

For further analysis of effects on performance and accuracy, multiple linear regressions with group, condition, and age as predictors were conducted for each timepoint.

Performance and accuracy over the separate blocks per session, was analyzed with a mixed ANOVA. Besides group and noise conditions as between variables, block number was included as a repeated within variable, condition as a between variable.

Due to lack of normal distribution paired Wilcox rank-sum tests were conducted to compare post-questions about stress-level, performance, annoyance, and distraction between the two noise conditions.

All tests of significance were conducted with the RStudio Software (Version 1.1.414) against a level of *α* = 0.05.

## Results

### Online survey

Three hundred forty-eight healthcare professionals (273 females) from the German-speaking part of Switzerland participated in the online survey (16 incomplete questionnaires). Mean age was *M* = 39.5 years (± 9.8, range 21 to 63). Besides 200 experts in critical care, 73 participants were in training to become experts, 44 were nursing professionals, 16 were senior physicians, and 15 were assistant physicians.

**Fig. 2:**
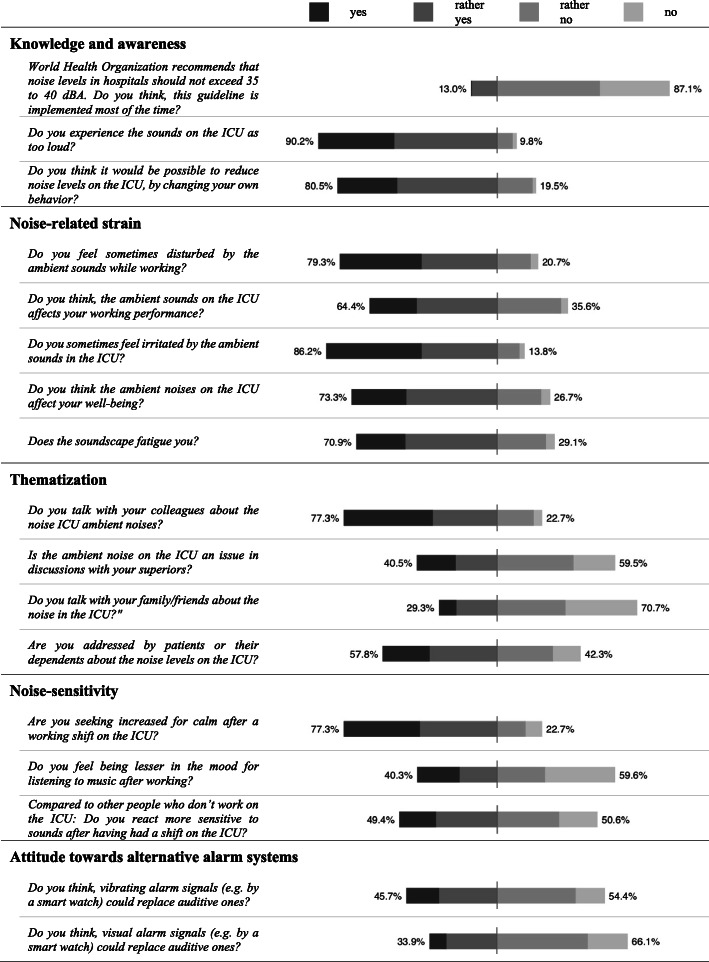
Online survey results. Percentages of “yes” and “rather yes” compared to “no” and “rather no” answers

Relative proportions of answers on the first 17 items of the online survey are given in Fig. 2. Mean irritation ratings and dBA estimations per noise source are indicated in Table 1. Pearson correlation between mean irritation ratings over all participants with mean dBA estimates was *r* (18) = 0.88, *p* < 0.001. Sound level estimations significantly contributed to prediction of irritation ratings (*β* = 0.05, *p* < 0.001). The predictor explained 77% of the variance (*R*^*2*^ = 0.771, *F* (1, 18) = 60.7, *p* < 0.01). Further irritating noise sources can be found in the supplement (Additional file 1).

### Experiment

Twenty-six healthcare professionals (21 females) and 27 controls (20 females) without previous working experience in a medical setting participated in the noise exposure experiment. Mean age was *M* = 33.2 (± 11.8, range 19-59), whereas on mean age of healthcare professionals was higher (*M* = 36.7 years, ± 9.67) than of controls (*M* = 29.9 years, ± 12.9).

Analysis of the pre-noise exposure questionnaire measurements did not show any significant differences between conditions or groups concerning sleep quality, strain, and alertness. Mean age of controls (*M* = 29.9, *±* 12.9) was significantly lower than mean age of the HCP group (*M* = 36.7, *±* 9.67), *t* (48.1) = 2.188, *p* = 0.03. Noise sensitivity scores did not differ between noise conditions (*F* (1, 49) = 1.84, *p* = 0.18) but did between the two groups (*F* (1, 49) = 11.76, *p* = 0.001). With a mean score of *M* = 50 (*±* 12.2), noise sensitivity of controls was significantly lower than of HCPs (mean of *M* = 61, *±* 10.8). Only the grouping variable (*β* = 10.1, *p* < 0.01) not the age variable (*β* = 0.13, *p* < 0.37) contributed significantly to prediction of noise sensitivity in a regression model (*R*^*2*^ = 0.20, *F* (2, 50) = 6.23, *p* < 0.01). When excluding controls, age as a predictor for noise sensitivity scores in HCPs reached significance (*β* = 0.46, *p* = 0.04, *R*^*2*^ = 0.17, *F* (1, 24) = 4.97, *p* = 0.04). The model including the duration subjects already worked in an ICU was significant (*β* = 0.54, *p* = 0.04, *R*^*2*^ = 0.16, *F* (1, 24) = 4.61, *p* = 0.04). Age and ICU experience strongly correlated with *r* (24) = 0.93, *p* < 0.001.

Mean 2-back task accuracy and performance per session are indicated in Table 2.

**Table 2:** Working memory task performance and accuracy per noise condition and sessions. The values represented are mean ± standard deviation

	Performance		Accuracy	
	ICU noise	Pink noise	ICU noise	Pink noise
Session 1	0.76 ± 0.13	0.78 ± 0.14	0.84 ± 0.10	0.84 ± 0.08
Session 2	0.82 ± 0.14	0.82 ± 0.17	0.85 ± 0.09	0.87 ± 0.12

In the linear regression, age was the only significant contributing factor to variance in accuracy of the first (*β* = −0.003, *p* < 0.01, *R*^*2*^ = 0.26, *F* (3, 49) = 5.85, *p* < 0.01) and second session (*β* = −0.004, *p* < 0.01, *R*^*2*^ = 0.28, *F* (3, 49) = 6.29, *p* < 0.01) as well as to variance in performance of the first (*β* = −0.008, *p* < 0.01, *R*^*2*^ = 0.46, *F* (3, 49) = 13.9, *p* < 0.01) and second session (*β* = −0.008, *p* < 0.01, *R*^*2*^ = 0.38, *F* (3, 49) = 9.88, *p* < 0.01). Neither the effects of group nor noise condition reached significance in one of the models. Mixed ANOVAs did not show any main effect of the grouping variable, noise condition, or block. Further, none of their interactions reached significance. Detailed values can be found in the supplement (Additional file 2). The results indicate that improvements in accuracy and performance over blocks do not differ between conditions or groups.

Results of post questionnaires comparing ICU and pink noise conditions are given in Table 3.

**Table 3:** Results of post-task questions about perceived strains by noise conditions

Questions	Noise	Mean ± SD	Median	*Z*	*p*	Sign
“How stressed did you feel during the task?”	Pink	47.1 ± 23.9	50	856.5	0.002	**
	ICU	61.6 ± 19.2	66.5			
“I experienced the background sound as too loud.”	Pink	1.96 ± 0.71	2	762	< 0.001	***
	ICU	2.87 ± 0.89	3			
“During the n-Back task, I felt distracted by the background sound.”	Pink	1.96 ± 0.79	2	640	< 0.001	***
	ICU	2.87 ± 0.95	3			
“I felt annoyed by the background sounds.”	Pink	1.83 ± 0.76	2	355	0.002	**
	ICU	2.37 ± 0.91	2			
“I was able to solve the task reliable and correctly.”	Pink	2.38 ± 0.77	3	107	0.007	**
	ICU	2.00 ± 0.77	2			

*SD* standard deviation, *sign* significance

*p < 0.05

**p < 0.01

***p < 0.001

## Discussion

### Awareness and perceived strain

The results of the online survey indicate that awareness of heightened sound levels is common among healthcare professionals. A majority of participants perceived the ICU as a loud environment and knew that sound levels could exceed WHO recommendations. Most participants stated that ICU ambient sounds could impair well-being and working performance, e.g., by inducing fatigue and irritation.

These findings are in line with data from Ryherd, Waye, and Ljungkvist who collected data of 47 HCPs with a similar questionnaire [13]. However, in the current survey, more participants reported to be affected by noise with a higher proportion of participants reporting having previously discussed the issue with colleagues or superiors. Thus, noise might be a matter of concern.

### Noise-induced annoyance

As expected, several ICU noises are perceived as irritating and annoying. There is evidence that the degree of annoyance does not solely depend on acoustical but also on non-acoustical features (e.g., unpredictability, timing) [2, 30]. Especially sudden and loud alarming signals and staff-generated noises seem to be the most annoying for patients [6] and for healthcare professionals [9, 31].

### Noise and stress

In the described experimental setting, self-rated stress levels were significantly higher after ICU noise versus pink noise exposure, indicating that healthcare professionals may perceive ICU-noises as stressful. These results are in line with other studies, which used self-ratings or physiological measures as stress indicators [9, 10]. It seems likely that sound levels might play a mediating rather than a causal role: better coping with stress may theoretically occur when noise levels are low [32]. Since long term effects of stress are highly relevant for physiological and mental health [1], physiological stress answers (e.g., measurements of skin conductance or heart rate variability) in HCPs should be examined and might be considered outcome factors in subsequent noise intervention studies.

### Cognitive and task performance

Ambient sounds in an ICU meet most criteria that are known to disturb working memory performance: They are discontinuous, have changing states, and often contain speech [19]—even though the results of the 2-back WMT did not show any significant changes of performance. This seems to contradict the results of Murthy et al. who assessed the effect of operating room soundscape on the cognitive efficiency of anesthetist [16]. Several factors might have led to this discrepancy: First, Murthy and colleagues did not use the same cognitive tests. Possibly, the 2-back task used in this study might be less sensitive to detect differences between respective conditions. Second, participants’ mean age and variation of age were lower in the study of Murthy et al. and thus, working memory performance might have varied to a smaller degree between subjects. Third, in Murthy and colleagues’ study, noise exposure was longer. Fourth, pink noise at a level of 35 dBA was used as a control background sound, whereas Murthy and colleagues conducted a silent control condition.

In addition, several studies that looked at job-specific task performance rather than at mental efficiency failed to show significant effects [33, 34]. Possibly, healthcare professionals adapted to noisy working conditions or ambient sounds on ICUs, hence their performance is simply not affected.

But even though clear evidence is missing, there are at least three reasons that may justify the assumption that noise exposure could impair performance: First, self-ratings of this and other studies indicate that a majority of ICU healthcare professionals perceive ambient noise as performance reducing [13, 14]. Second, annoyance is often a consequence of increased mental workload due to distractions and thereby a sign of reduced cognitive resources [19]. From this viewpoint, noise-induced annoyance could be linked to diminished working performance. Third, noise is a stressor and thus, over time, might reduce cognitive resources [35].

Again, the influence of noise on working memory and task performance might depend on the specific task and person [36]. Further, possible effects might be delayed: e.g., increased coping efforts during work could lead to diminished cognitive capacity and increased fatigue after work [37].

### Noise sensitivity

HCPs participating in the experimental noise exposure had significantly higher noise sensitivity scores than controls. Even though scores did not correlate with age in controls there was a correlation in HCPs. Since noise sensitivity correlated also with the duration subjects already have been working in an ICU, it can be assumed that duration might be a mediating factor between age and sensitivity scores. This would imply that the longer someone worked on an ICU, the more sensitive they react to noises. Noise-induced stress could accumulate over the years resulting in heightened sensitivity. Considering that at the same time lower noise sensitivity seems to improve the capacity to cope with noise-induced stress [36], noise reduction in ICUs could improve the mental and physical health of HCPs.

### Hospital politics

Though most online survey participants perceived ICU noises as burden and reported to discuss the concern with colleagues, only a minority reported that the topic was addressed with superiors (e.g., with hospital management). This discrepancy seems surprising. Hospital management might develop strategies to reduce noise: studies showed significant reductions in noise levels by floor planning, alternative equipment, and materials (e.g., sound absorbing materials) [38, 39] or by routine changes (e.g., quiet hours) and behavioral interventions [40–44]. Behavioral interventions and exchange of information should not only address health but also cleaning staff [45]. Though our survey shows that skepticism concerning alternative alarming systems might be common, new and more intelligent, self-prioritizing alarming systems which are removed from the patient bed with direct reporting to staff might be helpful in the future.

### Limitations

Our study has several important limitations that deserve discussion. First, generalization of the results might be challenged since some of the used questionnaires were not validated and results could be biased due to self-selection. Possibly, subjects were more willing to participate if they perceive noise as problematic. Further, all limitations of self-reporting or self-assessment (regarding the questionnaire) apply and the number of participants in the experimental setting is rather small. Second, sound pressure levels may vary strongly among ICUs and used equipment. Thus, the concern might not affect all healthcare professionals equally. Third, it is known that specific noise characteristics (e.g. volume level, pitch) have different effects on performance and well-being. In this study, a noise scenario including a wide range of sounds was used. Clearly, ambient noises (and therefore the specific noise characteristics) can vary strongly. Therefore, our results are not directly transferable to other ICU scenarios. Moreover, pink noise was used as a control condition because this is more realistic to achieve in an ICU setting than total silence. But in future work, it would be interesting to conduct a study with a third “silent control” condition. Fourth, our study—and many others in this field—used task-independent noise exposure, and thus, subjects could simply try to ignore the ambient sounds. Since in daily routine, HCPs have to pay attention to their surroundings, this approach suffers from reduced ecological validity and makes transfer of results difficult.

## Conclusion

Subjective assessments indicate that noise in ICUs may induce annoyance, with heightened stress levels, impaired well-being, and reduced performance being potential consequences. Even though this and other studies did not find significant effects of ICU noise exposure on cognition. Empirical evidence with objective and physiological measures is warranted.

## Supplementary information

**Additional File 1.** Further noise sources according to their categories.

**Additional File 2.** Results from the mixed ANOVAs of working memory performance and accuracy over three blocks separated per session.

## Data Availability

The dataset used and analyzed during the current study are available from the corresponding author on reasonable request.

## Abbreviations

ANOVA: Analysis of variance
CPR: Cardiopulmonary resuscitation
D-MEQ: Deutscher Morningness-Eveningness-Questionnaire
ECMO: Extracorporeal membrane oxygenation
HCP: Healthcare Professionals
ICU: Intensive Care Unit
OR: Operating room
SF-A/R: Schlaffragebogen A
WHO: World Health Organization
WMT: Working memory task
WNS: Weinstein Noise Sensitivity Scale
