# Supplementary material for: Effects of intensive care unit ambient sounds on healthcare professionals: results of an online survey and noise exposure in an experimental setting
Source: Intensive Care Med Exp. 2020 Jul 23;8:34. doi: 10.1186/s40635-020-00321-3 (PMC7376325; doi:10.1186/s40635-020-00321-3)
Supplement: Supplementary file 1 — Additional File 1. Further noise sources according to their categories. [file 40635_2020_321_MOESM1_ESM.pdf]

Equipment (continuous)	Alarms/ impulsive equipment noises
- Invasive cooling system<br>- Pneumatic stockings<br>- Automated bedpan<br>- Automated tap<br>- Cleaning machine (floor)<br>- Machine for arterial blood gas analysis (ABGA)<br>- Sequential compression devices (SCD-pump)<br>- Oscillator<br>- Pulse contour cardiac output (PICCO) Monitoring<br>- Oxygen moisturization<br>- Alternating pressure mattress<br>- High-flow apparatus (oxygen therapy): BIPAP (bilevel positive airway pressure) or CPAP (continuous positive airway pressure)<br>- Inductively coupled plasma (ICP) instruments<br>- Office chairs with roles<br>- Buzzing noises of machines	- Mattress<br>- Telemetry (especially false-alarms)<br>- Heating lamp<br>- Battery alarms (e.g. Bed)<br>- Resuscitation pager<br>- Service mobile phones<br>- Vacuum assisted closure-therapy<br>- Full face masks (e.g. Respironics)<br>- All alarms that cannot be turned off<br>- Alarms which are not quitted/ answered
**Human (equipment)**	**Human (speech, others)**
- Taking off gloves<br>- Shoes with wooden heels<br>- Computer of medical visit<br>- Computer keyboard<br>- Changing negative-pressure wound therapy-bandaging material<br>- Closing curtains<br>- Visitors which don't turn down their phones<br>- Pull chairs over the floor<br>- Open/ close laundry trollies<br>- Folding up/ down bed barrier<br>- "bed bow" falls on the floor<br>- Garbage bag changing<br>- Moving material trolleys<br>- Interventions (e.g. Placing jejunal probe)<br>- Hectic functioning during normal caring activities<br>- Cleaning floor with a mop (hectically, hitting objects)<br>- Closing/ opening doors	- Loud laughing<br>- Shouting<br>- Phoning<br>- Screaming (deliriant) patients, crying children<br>- Interposed questions of visitors during shift changes/ when reading into report<br>- Conversations across the room<br>- Announcements in all rooms<br>- Shift changes (physicians)<br>- Music or television
**Structural**	
- Airconditioning, ventilation system<br>- Environmental sound from outside (construction noise, ambulance car, helicopters, delivery vehicles)<br>- Opening/ closing doors of isolation rooms<br>- Taps with strong jets inside patient rooms<br>- Coffee machine<br>- Rooms with two or more beds<br>- Noises from corridor (shift supervisors, administration desk)	
