# Supplementary material for: Effects of intensive care unit ambient sounds on healthcare professionals: results of an online survey and noise exposure in an experimental setting
Source: Intensive Care Med Exp. 2020 Jul 23;8:34. doi: 10.1186/s40635-020-00321-3 (PMC7376325; doi:10.1186/s40635-020-00321-3)
Supplement: Supplementary file 2 — Additional File 2. Results from the mixed ANOVAs of working memory performance and accuracy over three blocks separated per session. [file 40635_2020_321_MOESM2_ESM.pdf]

	Session 1					Session 2				
**Performance**	*Effect*	*MS*	*df*	*F*	*p*	*Effect*	*MS*	*df*	*F*	*p*
Noise	0.02	0.02	1	0.87	.353	0.00	0.00	1	0.00	.987
Group	0.03	0.03	1	1.16	.282	0.06	0.06	1	2.09	.150
Block	0.08	0.04	2	1.73	.181	0.05	0.03	2	0.97	.381
Noise x Group	0.00	0.00	1	0.02	.881	0.06	0.06	1	2.32	.130
Noise x Block	0.00	0.00	2	0.07	.933	0.01	0.01	2	0.22	.805
Group x Block	0.01	0.00	2	0.11	.897	0.00	0.00	2	0.05	.955
Noise x Group x Block	0.01	0.00	2	0.20	.816	0.00	0.00	2	0.08	.920
Residuals	3.22	0.02	144			3.85	0.03	144		
**Accuracy**	*Effect*	*MS*	*df*	*F*	*p*	*Effect*	*MS*	*df*	*F*	*p*
Noise	0.00	0.00	1	0.03	.855	0.01	0.01	1	1.08	.300
Group	0.00	0.00	1	0.44	.510	0.01	0.01	1	0.50	.483
Block	0.02	0.01	2	0.87	.420	0.02	0.01	2	0.90	.408
Noise x Group	0.01	0.01	1	1.15	.286	0.02	0.02	1	1.89	.172
Noise x Block	0.01	0.01	2	0.75	.476	0.00	0.00	2	0.18	.834
Group x Block	0.01	0.00	2	0.33	.723	0.00	0.00	2	0.01	.990
Noise x Group x Block	0.00	0.00	2	0.22	.800	0.00	0.00	2	0.07	.931
Residuals	1.42	0.01	144			1.82	0.01	144		

Effect Main and Interaction Effects, *MS* Mean squares, *df* Degrees of freedom
